# Laser-triggered combination therapy by iron sulfide-doxorubicin@functionalized nanozymes for breast cancer therapy

**DOI:** 10.1186/s12951-021-01023-y

**Published:** 2021-10-27

**Authors:** Shipeng Ning, Yang Zheng, Kun Qiao, Guozheng Li, Qian Bai, Shouping Xu

**Affiliations:** 1 Department of Breast Surgery, Guangxi Medical University Cancer Hospital, Nanning, 530000 China; 2 Department of Breast Surgery, Harbin Medical University Cancer Hospital, 150 haping Road, Nangang District, Harbin, 150000 China; 3 Department of Anesthesiology, The Second Affiliated Hospital of Zhengzhou University, Zhengzhou, People’s Republic of China

**Keywords:** Breast cancer, Gas therapy, Chemotherapy, Nanozyme, Fenton reaction

## Abstract

**Background:**

The use of magnetic nanozymes (NZs) with the ability to synchronize gas therapy through photodynamic and chemotherapy in the treatment of breast cancer has received much attention.

**Results:**

Hence, in this study, we designed a bovine lactoferrin-coated iron sulfide NZs containing doxorubicin (abbreviated as: FeS-Dox@bLf NZs) by wet-chemical synthesis method. Then, the physicochemical characteristics of synthesized NZs were explored by several methods. Also, the level of Fe^2+^, H_2_S and Dox releases from FeS-Dox@Lf NZs. Also, the cytotoxic effects of FeS-Dox@Lf NZs were investigated by cellular assays. After intravenous injections of NZs and laser irradiation, significant effects of FeS-Dox@Lf NZs on mice weight and tumor status were observed. Afterwards, not only the distribution of Dox in the body was examined by fluorescent, but also the time of Fe clearance and the amount of Dox and Fe retention in vital tissues were determined. The findings confirm that FeS-Dox@Lf NZs, in addition to targeted drug distribution in tumor tissue, resulted in superior therapeutic performance compared to free Dox due to reduced Dox side effects in vital tissues, and increased level of free radicals in 4T1 cells.

**Conclusion:**

Overall, FeS-Dox@Lf NZs with the ability to synchronize chemotherapy and gas therapy raised hopes for more effective treatment of breast cancer.

**Graphic abstract:**

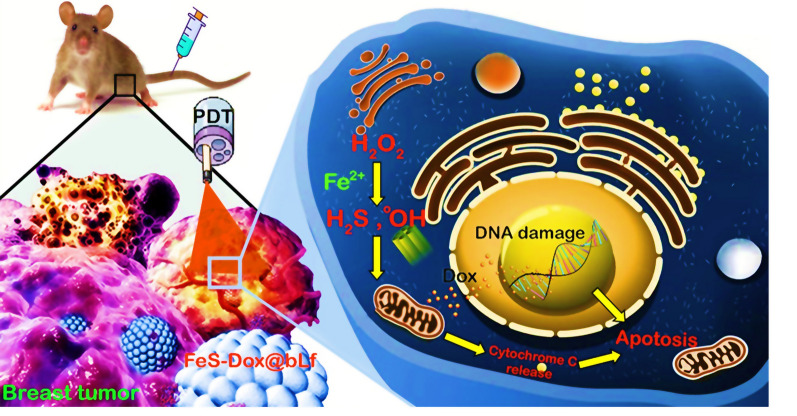

## Introduction

Breast cancer is one of the most challenging diseases in women that the use of new techniques based on nanoplatforms with less invasiveness has increased the hopes of treatment [1, 2]. In this regard, the use of magnetic nanoparticles (NPs) with enzyme-like behaviors for producing radicals [3, 4] along with the provision of imaging [5], photothermal therapy (PTT) [6], photodynamic therapy (PDT) [7] and chemotherapy [8] have received much attention. The enzymatic-like activity of magnetic NPs induces apoptosis by persuading radicals in cancerous cells through damage of fat, protein and DNA [9, 10]. For this purpose, several papers have been published based on iron nanozymes (Fe NZs) that show the formation of radicals through the Fenton reaction on hydrogen peroxide (H_2_O_2_) inside and outside of cells [11–13]. However, the results demonstrate that the amount of H_2_O_2_ in cancerous cells, especially breast cancer, is not high enough to generate reactive oxygen species (ROS) by Fe NZs [4, 14, 15]. On the other hand, several results exhibit that cancerous cells use enzymatic and non-enzymatic antioxidant defense systems to inhibit the generation and accumulation of radicals [16, 17]. Therefore, development of free radicals-based therapeutic platforms to control breast cancer cells have received a great deal of interest, recently.

Gas therapy through hydrogen, nitric oxide (NO) and hydrogen sulfide (H_2_S) are modern and effective methods in medical activities for antibacterial [18], cardiovascular [19], neurological [20], anti-inflammatory [21] and anti-cancer activities [22, 23]. Conventionally, H_2_S and NO gases have been reported in the human body, especially in the mitochondria, which can enhance their use in medical practice [24]. The reports reveal that H_2_S kills breast cancer cells by stopping the cell cycle through damage to mitochondria and miRNA [25, 26]. On the other hand, H_2_S inhibits catalase activity dramatically [27]. Because, H_2_S donors induce toxicity through the rapid release of gas, some bio-alternatives approaches are recommended.

Furthermore, the lack of a completely successful strategy in the application of iron sulfide (FeS) NZs, the use of anti-cancer drugs such as doxorubicin (Dox), and the formation of coatings on NZs to reduce toxicity and improve targeting can increase the likelihood of success. Despite the ambiguities in the use of Dox in the treatment of breast cancer, several reports indicate that Dox causes breast cancer cell death by increasing ROS and overexpression of Fass, Bax and caspase-3 [6, 8, 28, 29]. On the other hand, various results show that the challenge of Dox toxicity on heart cells [8, 30] and on the other hand the drug resistance of cancer cells by nanocarriers with bovine lactoferrin (bLf) coating is significantly reduced [31, 32].

In this study, not only the synthesis of FeS-Dox@bLf NZs was investigated to synchronize chemotherapy and gas therapy, but also the toxicity and drug loading along with the level of H_2_S and radical release were investigated. Also, in this study, the condition of the tumor, the amount of Dox and Fe retention in non-target tissues, and possible damage in non-target tissues were investigated. In fact, we aimed to provide a biocompatible platform for integrating therapeutic activities with minimally invasive approach in order to reduce the therapeutic resistance of breast cancer cells and their effective treatment.

## Material and methods

### Materials

Fe(NO_3_)_3_·9H_2_O, chloroacetic acid, NaOH, thiourea, 1-ethyl-3-(3-dimethylaminopropyl) carbodiimide (EDC), *N*-hydroxysuccinimide (NHS), dimethyl sulfoxide (DMSO), methylene blue (MB), and polyvinylpyrrolidone were prepared from the Merck (Germany). Cell culture materials were obtained from Gibco (Scotland).

### Synthesis of FeS@bLf NZs

Initially, a three-core Fe was provided to produce FeS NZs. In this regard, a chloroacetate solution produced from ClCH_2_COOH (5 g, 54 mmol) and NaOH (2.2 g, 55 mmol) in 100 mL of water was gradually added to 11 mL of Fe(NO_3_)_3_·9H_2_O (11 g, 27.5 mmol) solution. After 3 h, the red solution was filtered and kept at 21 °C for 2 weeks. Then, the precipitated red crystals were washed with cold methanol and air-dried. The resulting red crystals (420 mg; 0.45 mmol) were dissolved in 35 mL of water, followed by addition of 10 mL of aqueous solution containing 110 mg (1.45 mmol) of thio-urea and 700 mg of polyvinylpyrrolidone by stirring. Afterwards, the clear solution was kept into an autoclave with 150 °C (increase by 5 °C per min) for 14 h. Finally, the FeS NZs was gathered via centrifugation (7000*g* in 10 min), washed several times with methanol, and air-dried. The FeS NZs synthesis process was performed under oxygen-free conditions in a nitrogen atmosphere, as well as in oxygen-free water produced by boiling the solution and cooling it under a stream of free nitrogen.

Then, a mixture of 300 μL of 15 mg/mL EDC, 300 μL of 15 mg/mL NHS, and 10 μL of 1 M NaOH were used to organize carboxyl linkers on the FeS NZs for creating bLf. In this field, 1 mg of FeS NZs was sonicated for 20 min in 300 μL of the solution prepared. Then, surplus EDC and NHS was removed using PD-10 column. Afterwards, a defined amount of bLf was added to the activated FeS NZs for 24 h at room temperature. Finally, centrifugation (5000*g*, at 5 min) and DI water were applied to detach the FeS@bLf. After the production of FeS@bLf NZs, 3 mg of FeS@bLf NZs were mixed with 3 mL of DMSO solution containing 3 mg of Dox for loading the Dox into the NZs. Then, the NZs were dried under vacuum for 24 h. Ultimately, PBS was applied to wash the NZs 3 times to remove the discharged Dox via shaking for a 1 min.

### Characterization of FeS-Dox@bLf NZs

The FeS-Dox@bLf NZ morphology was considered using a scanning electron microscope (SEM, 250 FEG Quanta). As well, transmission electron microscope (TEM) image was provided on a high-resolution TEM (Carl Zeiss, Oberkochen, Germany) at an accelerating voltage of 200 kV. Moreover, the hydrodynamic size and zeta potential of FeS-Dox@bLf NZs were measured by Zetasizer Nano-ZS (Malvern, UK). 10 μL of FeS-Dox@bLf NZs was dispersed in 10 mM PBS solution. Furthermore, infrared spectra with a Nicolet model 759 FTIR spectrometer were evaluated in wavenumbers from 4000 to 500 cm^−1^ using FTIR spectroscopy (Perkin Elmer Inc., Wellesley, MA, USA).

### Hydroxyl radical formation catalysed by FeS-Dox@bLf NZ

The photocatalytic degradation of MB by FeS-Dox@bLf NZ was performed at 22 °C using 40 mL MB aqueous solution (6 × 10^–6^ M) containing 30 mM NaHCO_3_ and 11 mM H_2_O_2_, and 20 mg of FeS-Dox@bLf NZ with continuous stirring in a dark flask for 30 min. Then, suspension was irradiated with the tungsten halogen lamp (100 W). During the determined time periods, 3 mL of the aliquot was removed from the mixture and centrifuged and the dye concentration was measured with an UV–Vis spectrophotometer.

### Fe^+^, H_2_S and drug release

To determine the level of Fe^+^ released, 450 μL of FeS-Dox@bLf NZ (6 mM) was added into a dialysis bag with PBS buffer (60 mL, 12 mM) at pH 6.5 and 7.2. The solutions were maintained at 37 °C and 3 mL was collected for analysis at 0, 0.5, 1, 2, 5, 8, 16, 24, 48, and 72 h. The Fe elements were quantified using atomic absorption spectrophotometry. Likewise, for evaluating H_2_S, 450 μL of FeS-Dox@bLf NZs (6 mM) was added into a dialysis bag with HEPES buffer (60 mL, 12 mM) at pH 6.5 and 7.2. Then, 2 mL of the solution was gathered for H_2_S analysis at 0, 1, 2, 5, 10, 20, 30, 40, 50, 60 min. H_2_S concentration was evaluated using a standard MB method based on the description of Lin et al. [33].

To investigate the possibility of in vitro drug release from NZs, the FeS-Dox@bLf NZs were incubated at 37 °C for 900 min in PBS at different pH (6.5 and 7.2). Generally, certain amounts of NZs were diffused in 10 mL of PBS and put in the dialysis bag (MWCO 3500). Afterwards, the bag was dialyzed against 45 mL of the same buffer at 120 rpm. At designated times, 6 mL of solution the dialysis bag was withdrawn for the investigation via absorbance at 490 nm and was replaced by an equal volume of the same buffer. The Dox cumulative release was assessed based on the following Eq. (1):

1 $${\text {Cumulative drug release}} \left(\%\right)=\frac{6 \times {\sum }_{\mathrm{i}-1}^{\mathrm{n}-1}{\mathrm{C}}_{\mathrm{i}}+{\mathrm{V}}\times {\mathrm{C}}_{\mathrm{n}}}{\text {weight of Dox on FeS}@{\mathrm{bLf}}} \times 100,$$

where, *C*_*i*_ and *C*_*n*_ refer to the of Dox concentration at time *i* and *n*, and V was the volume of the buffer in the drug release experiment, respectively.

### In vitro assays

The 4T1 cells were seeded in DMEM medium with 10% FBS, 100 U/mL of penicillin, and 100 μg/mL of streptomycin and preserved in an incubator with 5% CO_2_ at 37 °C and 95% humidity.

#### In vitro combination therapy

The 4T1 cells were cultured at a density of 3 × 10^5^ cells per well into a 96-well plate and then incubated for 12 h at 37º C with 5% CO_2_. After 12 h, the 4T1 cells was treated and incubated for 24 h (main incubation) with different concentrations of free Dox (5, 10, 15, 20, and 25 μg/mL), FeS@bLf NZs and FeS-Dox@bLf NZs (12.5, 25, 37.5, 50 and 62.5 μg/mL) without or with laser irradiation (150 J/cm^2^ fluence and 100 mW/cm^2^ irradiance) for 7 min in 8th hour after main incubation. In the following, the MTT assay was performed at 570 nm.

#### Cellular concentration of hydroxyl radical and H_2_S

To evaluate the level of hydroxyl radical in cells based on the report of LeBel et al. [34], the 4T1 cells (5 × 10^5^ cells per well) were cultured, treated by FeS@bLf NZs (12.5, 25, 37.5, 50 and 62.5 μg/mL), and incubated for 24 h. During the incubation, the 4T1 cells were treated by laser irradiation for 7 min in the 8th hour after incubation. Briefly, 4T1 cells were collected and loaded with 30 μM the fluorogenic probe 2,7-dichlorofluorescin diacetate (DCFH-DA) at 37 °C for 30 min in DMEM medium without serum. The working volume DCFH-DA was supplemented proportionately according to the total cell numbers. After PBS washes, the DCF fluorescence released from DCFH-DA in the 4T1 cells was evaluated by a flow cytometer (CytoFLEX, Beckman Coulter, Brea, CA, USA) at an excitation wavelength of 500 nm.

In order to investigate intercellular H_2_S according to reports of Peng et al. [35], 5 × 10^5^ 4T1 cancer cells per well were cultured into 6-well plates for 12 h. After incubation, Washington State Probe-1 (C_33_H_21_NO_6_S_2_) (50 μM) was applied as H_2_S probe for 30 min and then treated with 12.5, 25, 37.5, 50 and 62.5 μg/mL of FeS@bLf without or with laser irradiation in 8th hour along with 200 μM of Na_2_S. Afterwards, supplemented HEPES containing 100 μM of CTAB (pH 7.4). After 10 min, samples were visualized immediately by a fluorescence microscope with a 465/515 nm and an excitation/emission filter set (ECLIPSE, TE2000-S, Nikon).

#### Apoptosis and ROS assays

To evaluate of apoptosis in the presence of free Dox (20 µg/mL), FeS-Dox@bLf NZs and FeS-Dox@bLf NZs + Laser (50 µg/mL), flow-cytometry was used. The 4T1 cells (5 × 10^5^ cells per well) were treated with drugs and NZs with (in 8th hours of main incubation) or without laser irradiation in for 24 h. Then, the cells were gathered with 3000*g* centrifugation at 4 °C for 3 min, and washed with PBS. Then, the 4T1 cells were resuspended in 100 μL per tube of containing Annexin V binding buffer (HEPES buffer: 0.1 M, NaCl 1.4 M, CaCl_2_ 25 mM, pH 7.4). In the following, 2 μL of Annexin-V was added to the cells solution and retained in dark for 15 min at 21 °C. Afterwards, 400 μL of the binding buffer and 5 μL of 50 μg/mL propidium iodide were added to the solution and reserved in ice. Finally, samples were analysed by BD FACSCalibur Flow Cytometer.

Similar to ^•^OH assay in Sect. 2.6.2, to determine intracellular ROS in the presence of free Dox (20 µg/mL), FeS-Dox@bLf NZs and FeS-Dox@bLf NZs + Laser (50 µg/mL), 5 × 10^5^ 4T1 cancerous cells per well were cultured into 6-well plate for 24 h. Then, the fluorescent intensity based on DCF measurement was evaluated.

### In vivo assays

For in vivo studies, 40 mice in 5 groups were used. For this purpose, 7-weekly female mice, weighing 25.6 ± 1.3 g were kept at 25 °C with 12 h of light, 55% humidity and free access to water and feed. The cultured 4T1 cancerous cells (1 × 10^6^ cell) according to Sect. 2.6 were injected subcutaneously (150 μL) at the end of the mammary gland on the right side of the mice. After the tumors reached a size of 144.1 ± 2.4 mm^3^, mice were treated 6 times, every 4 days (0, 4, 8, 12, 16, 20 days) with free Dox (10 mg/kg) and FeS-Dox@bLf NZs (20 mg/kg) with and without laser irradiation. The amount of Dox injection in free and FeS-Dox@bLf NZs conditions was equal.

#### Body weight and tumour size

The mice were orderly monitored for abnormal behaviour and were weighed every four days. Also, to measure the tumor volume (TV), the mice were evaluated in each measurement by the digital Vernier Caliper (Mitutoyo, Japan) based on Eq. (2):

2 $$TV (mm^{3}) = 1/2 \times (length \times width^{2}).$$

Furthermore, mice were sacrificed on day 18, and the tumours were collected and weighed.

#### Photodynamic therapy (PDT)

For PDT assay, the tumors were illuminated every four days and 8 h after free drug and FeS-Dox@bLf NZs injections, through the skin surface with spot diameter of 10 mm for 7 min with light of a 150 J/cm^2^ fluence and 100 mW/cm^2^ irradiance by applying a diode laser. Light with a wavelength of 630 nm to the entire tumor was set up to prevent possible damage.

#### Dox distribution

For studying the Dox concentration in main organs including heart, spleen, liver, lung and breast tumor tissues, the samples were collected, washed with cold saline and dried. Then, tissues were homogenized in an acetonitrile and water (50:50) mixture for liquid–liquid phase extraction explained by Zhang et al. [36].

Fluorescent imaging was used to investigate the bio-distribution of the Dox in the whole body and main organs. For this purpose, to determine the distribution of the Dox in the whole body, after 8 h from drug injection, the mice were anesthetized and fluorescence imaging was performed with excitation 485 nm and emission 590 nm. Likewise, to image the Dox distribution in vital organs, mice were sacrificed in 8 h after injection and their vital organs were collected and after washing with 0.9% NaCl, ex vivo fluorescence was used with the same device settings.

#### Excrete and distribution of Fe

In order to evaluate the concentration of excreted Fe via urine and faces, the animals were placed in metabolic glass cages. Faecal and urinary samples were collected 48 h and Fe concentration was determined on days 0 (to determine the background level), 8 and 16 days after FeS-Dox@bLf NZs administration. The amount of Fe in the samples was determined using atomic absorption spectrophotometry. Moreover, to evaluate the Fe distribution in tissues of tumor, heart, lung, spleen and liver, microwave digestion technique with mixture HNO_3_:HClO_4_ (5:1) was applied. Then, 0.2 g of digested samples was diluted with 50 mL of deionized water, and finally Fe elements were measured by atomic absorption spectrophotometry.

#### Histological assay

For histopathological evaluation of major organs including heart, liver, spleen, lung, and tumor, the mice were sacrificed and their organs were collected at the end of experiments. Formaldehyde (10%) was used to fix the tissue samples and then continued by passage and embedding in paraffin. To perform the hematoxylin and eosin (H&E) staining operation, paraffin blocks were partitioned by 3 μm thickness. Slides were studied with the microscopic (Olympus microscope).

### Statistical analysis

Statistical analysis was carried out with one-way ANOVA followed by least square means and statistical differences were measured at level of ^*^P < *0.05*, ^**^P < *0.01* and ^***^P < *0.001.*

## Results

### FeS-Dox@bLf NZs characterization

**Fig. 1:**
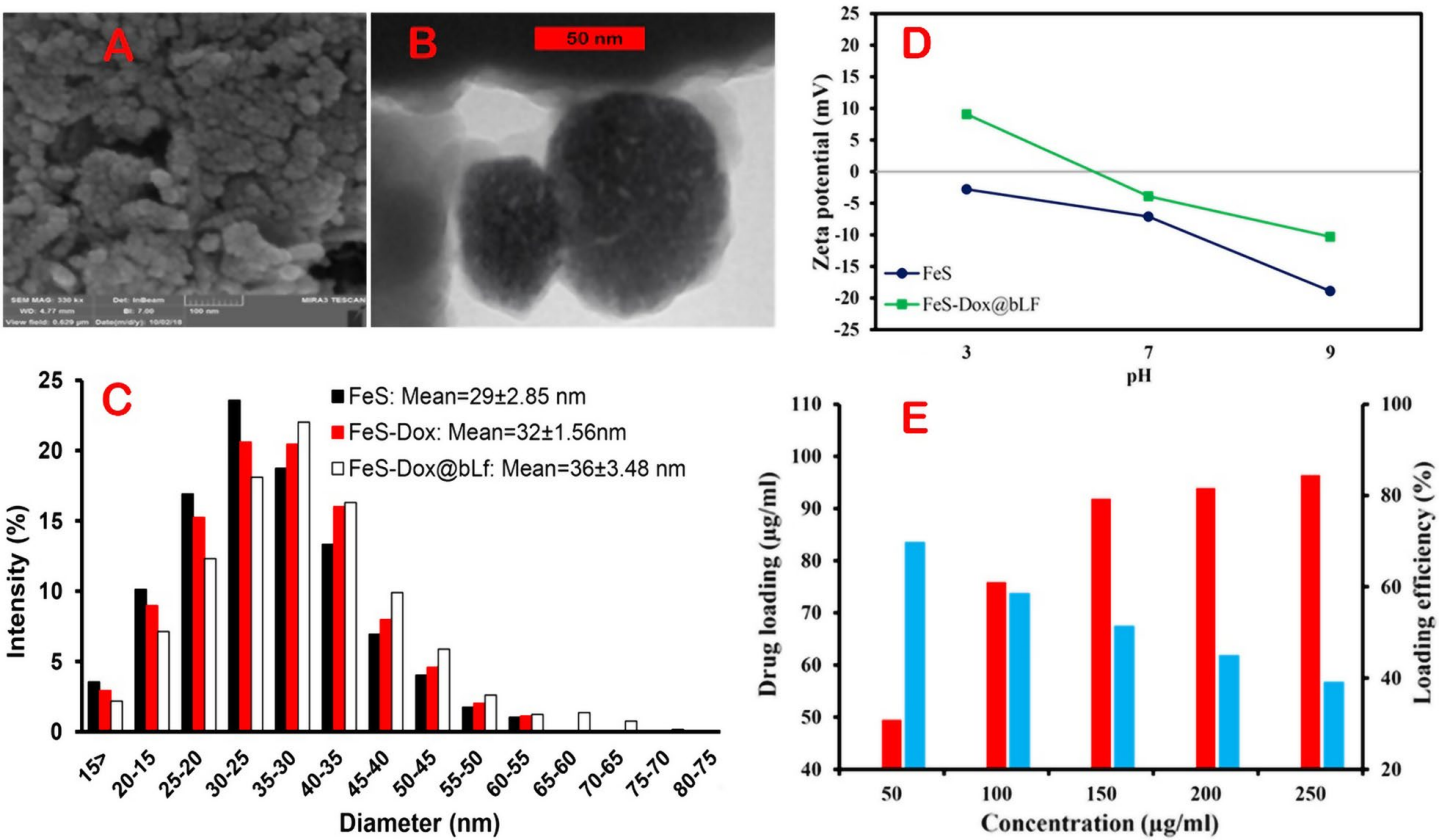
SEM (**A**) and TEM (**B**) images of FeS-Dox@bLf, (**C**) FeS, FeS-Dox and FeS-Dox@bLf sizes distribution determined by DLS, (**D**) Dependence of the zeta potential on the pH of the solution, (**E**) drug loading and its efficiency

The morphological properties of the FeS-Dox@bLf NZs produced were initially investigated by SEM and TEM. As shown in Fig. 1A, the FeS-Dox@bLf NZs are spherical with a size of 35 nm, and also the Lf coating covers them well. The SEM (Fig. 1A) and TEM (Fig. 1B) images show the relatively normal distribution of FeS-Dox@bLf NZs during synthesis. DLS results also show that the particle size of FeS varies from 5 to 70 nm with a polydispersity index (PDI) of 0.114, whereas drug loading increased the size of NPs to 7–75 nm with a PDI of 0.289 (Fig. 1C). Then, particle size diagram obtained by the DLS method in Fig. 1C shows that the size of FeS-Dox with dimensions of 7–75 nm in the presence of bLf increased to 10–80 nm with A PDI of 0.402. Therefore, the formation of bLf coating on FeS-Dox can be confirmed by increasing the particle size to at least 5 nm. The zeta potential data in Fig. 1D indicates that the NPs have zeta potentials of + 9.1, − 3.9 and − 10.3 mV at pH 3, 7 and 9, respectively, which can be part of the surface charge related to amino groups. Whereas, the zeta potential values for Fe NPs at pH 3, 7 and 9 were − 2.8, − 7.1 and − 18.9, respectively. Thus, not only the zeta potential confirms the presence of protein on Fe NPs, but also this data confirms the adequate colloidal stability of Fe NPs in biological and environmental applications. In this regard, the results of FT-IR on FeS, Dox, bLf and FeS-Dox@bLf confirm the loading of drug and bLf on the NZs (Data not shown).

The results of drug loading capacity (%) revealed that with rising Dox concentration, despite the constant concentration of FeS@bLf NZs (250 µg/mL), the amount of drug loading in NZ meaningfully increases (Fig. 1E). While, the loading efficiency (%) decreases with rising Dox concentrations. This profile proposes that the highest Dox loading in FeS@bLf NZs can be attained in the range of 100 μg/mL with an efficiency of ~ 58%.

**Fig. 2:**
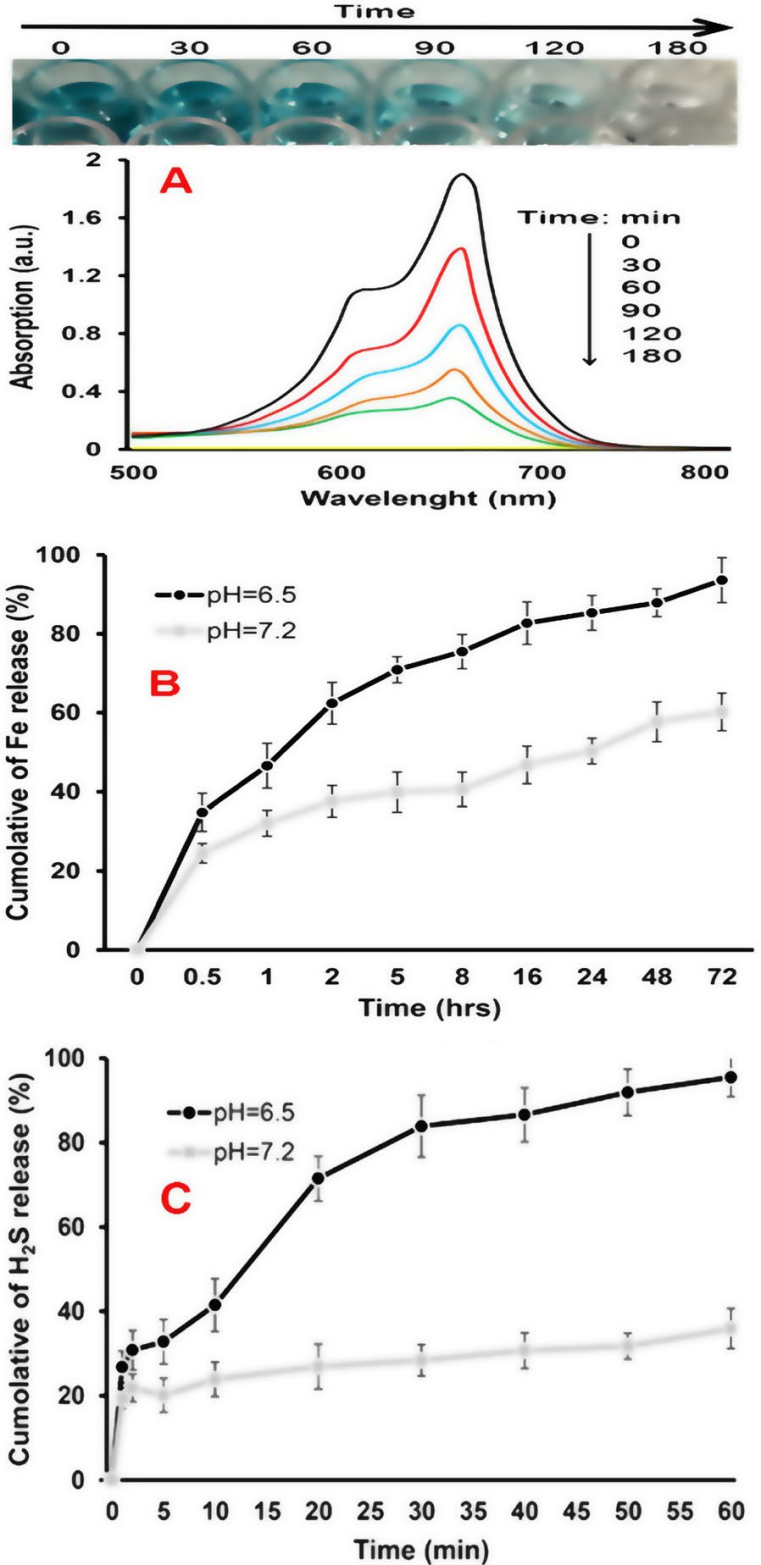
**A** Photographs of solutions and time dependent absorbance spectral change of aqueous solution of MB catalyzed by 20 mg FeS-Dox@bLf NZs adding to H_2_O_2_ (9 mM)/NaHCO_3_ (25 mM) solution under light irradiation (tungsten) for 180 min. **B** Levels of Fe^+^ released from FeS-Dox@bLf in PBS buffer of pH 6.5 and 7.2. **C** Levels of H_2_S gas released from FeS-Dox@bLf NZs at HEPES buffer of pH 6.5 and 7.2

The metallic NZs organize the catalysts for Fenton reaction in the blood, which can use H_2_O_2_ in tumors to produce ^•^OH. In order to explore the peroxidase-like behavior of FeS-Dox@bLf NZs, after adding NZs to a solution containing MB and H_2_O_2_ for 180 min, it was found that FeS-Dox@bLf NZs change blue color and gradually reduce the peak in the range of 600–700 nm by digesting MB (Fig. 2A). Color change with reducing MB confirms the time-dependent enzymatic activity of FeS-Dox@bLf.

### Fe^2+^, H_2_S and Dox release

To evaluate the degradability of FeS-Dox@bLf NZs in blood and tumor tissue, NZs were examined in aqueous medium with pH 6.5 and 7.2. As shown in Fig. 2B, FeS-Dox@bLf NZs not only decompose faster in an acidic environment, but also release higher Fe^2+^. For example, after 8 h, the level of Fe^2+^ released in the acid medium was higher than 75.5%, while the level of Fe^2+^ released in the neutral medium was up to 40.6%. This result indicates the possibility of Fe^2+^ release in 4T1 tumor cells.

Likewise, to survey H_2_S release, FeS-Dox@bLf NZs were dispersed in PBS at pH 6.5 and 7.2 for 60 min. The results of Fig. 2C shows a significant increase in H_2_S release by changing the acidity of the environment from neutral to acidic. As expected, FeS-Dox@bLf NZs in an acidic environment H_2_S release faster and more than in a neutral medium in line with the release of Fe^2+^. Therefore, the release of H_2_S dependent on environmental acidity by FeS-Dox@bLf NZs allows the intelligent treatment and diagnosis of cancerous tumors.

As well, by examining the release of the drug at pH 6.5 and 7.2 at 37 °C, it was determined that the Dox release from the FeS-Dox@bLf NZs based on Table 1 follows a time-dependent diffusion profile. The results indicated that the drug release rate in pH > 7 due to the function of bLf is higher than neutral (83.3 vs. 46.4 during 900 min). Despite the burst release of the Dox from FeS-Dox@bLf NZs, the rate of drug release over time is relatively constant and increasing. Above 80% Dox release from FeS-Dox@bLf NZs in acidic condition is a very good potential for drug release in cancerous tissues with pH 6–6.6.

**Table 1:** Cumulative percentage of Dox release at 37 °C at different pH

Time (min)	pH 6.5	SE	pH 7.2	SE
0	0	0	0	0
6	15.6	1.78	5.9	1.15
15	31.2	2.69	8.4	1.08
30	42.1	2.29	16.3	1.69
60	50.7	3.31	24.1	2.07
90	55.5	4.35	28.5	2.15
180	62.5	3.38	34.6	3.06
360	71.1	5.01	39.7	3.25
540	76.6	4.52	42.8	3.07
720	80.8	3.69	44.9	3.76
900	83.3	4.59	46.4	2.99

### In vitro assays

**Fig. 3:**
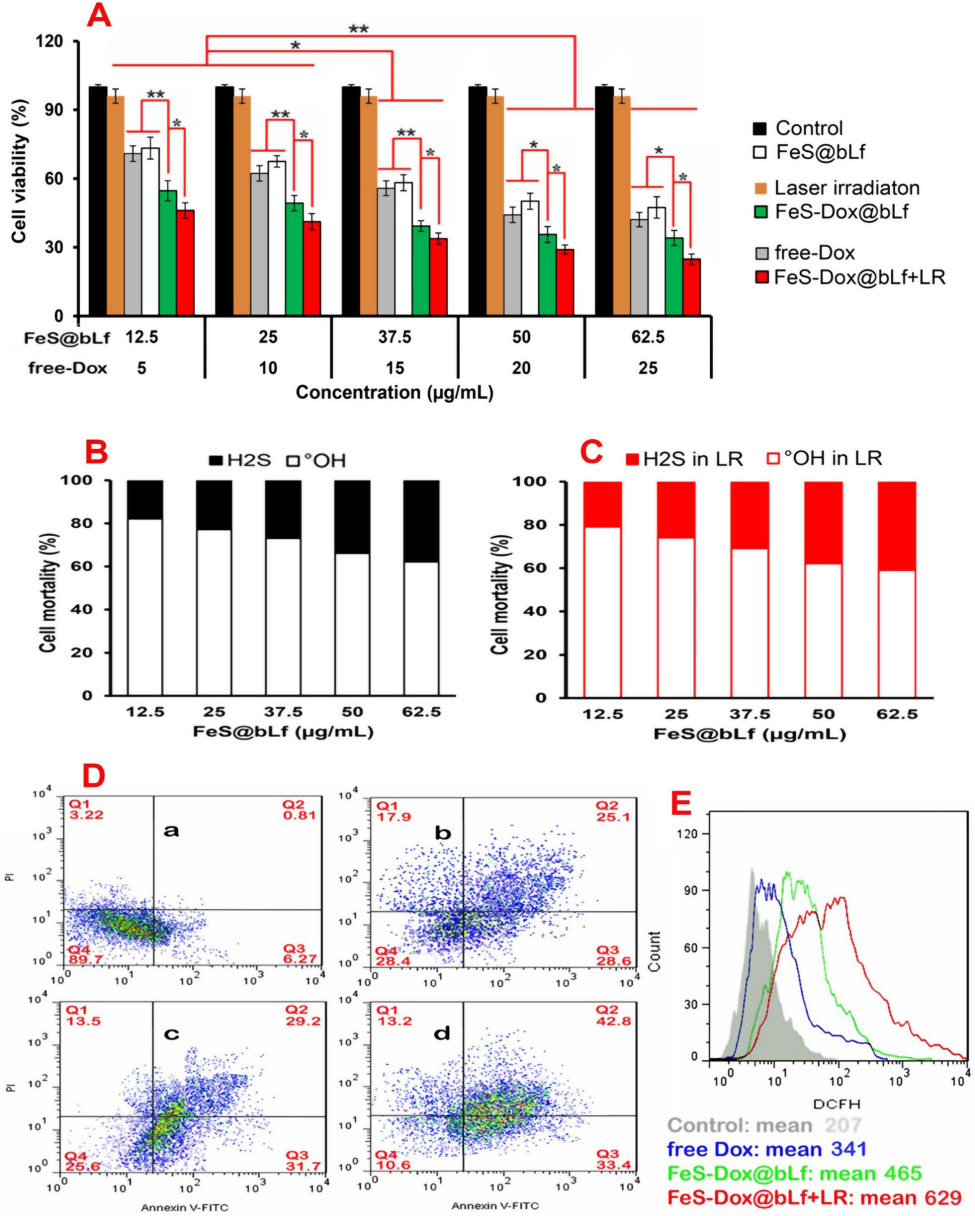
**A** Cytotoxicity test of control, laser irradiation, free Dox, FeS NZs, and FeS-Dox@bLf NZs with or without laser irradiation (LR), on 4T1 cells by MTT assay. Mortality of 4T1 cancerous cells triggered by ^•^OH radicals and H_2_S gas after treated with FeS@bLf without (**B**) or with laser irradiation (LR) for 24 h (**C**). **D** Two-dimensional contour density plots of 4T1 cells obtained by flow cytometry-based assays. a: Control, b: free Dox, c: FeS-Dox@bLf NZs, and d: FeS-Dox@bLf NZs + LR. Cell necrosis and apoptosis were measured using propidium iodide (PI) and Annexin V-FITCH dyes, and (**E**). The effects of a: Control, free Dox, FeS-Dox@bLf NZs, and FeS-Dox@bLf NZs + LR NZs on the ROS production. Statistical differences were measured at level of ^*^P < *0.05*, and ^**^P < *0.01*

Examination of free drug or NZs toxicity on 4T1 cancerous cells based on MTT technique revealed that free Dox, FeS@bLf, FeS-Dox@bLf NZs and FeS-Dox@bLf NZs + laser have a negative effect on 4T1 cells (Fig. 3A). Despite the high toxicity of FeS-Dox@bLf NZs on 4T1 cancer cells, the results of Fig. 3A shows that there is no significant difference between the toxicity of free Dox and FeS@bLf at different concentrations on 4T1 cancer cells. On the other hand, the use of FeS-Dox@bLf NZs + laser exposed the most toxicity for 4T1 cancerous cells compared to other groups. Overall, the results revealed that increasing the concentration of NZs and the free drug increased their toxicity.

Since part of the therapeutic effects of FeS-Dox@bLf NZs in this study are based on H_2_S and ^•^OH release, the death rate of 4T1 cancerous cells death based on H_2_S and ^•^OH were investigated and reported in Fig. 3B, C. As seen, the most toxic effects of FeS@bLf NZs on 4T1 cells death are based on ^•^OH concentration, which with increasing FeS@bLf NZs concentration, the ^•^OH effect is constantly reduced and added to the H_2_S. On the other hand, the use of lasers on FeS@bLf NZs in this study showed that ^•^OH accounts for a large proportion of cancer cell death (^*^P ≤ 0.05). It is generally observed that the effects of ^•^OH are much more powerful than H_2_S production. However, laser irradiation of NZs increases the H_2_S activity in them, which indicates an increase in S^2−^ release and induction of more enzymatic reactions with the released Fe^2+^.

In order to confirm the greater toxicity of FeS-Dox@bLf NZs with and without laser therapy compared to free Dox in therapeutic activities, apoptosis and ROS experiments (Fig. 3D, E) demonstrated that FeS-Dox@bLf NZs with and without laser provided the highest toxicity and cancer cell death compared to free Dox. For instance, apoptotic cells percentage in 4T1 cancer cells after 24 h indicated that FeS-Dox@bLf NZs, compared to the free Dox group, increased the induction of apoptosis in Q2 (early apoptosis) and Q3 (late apoptosis) quarters from 25.1 to 29.2% (^***^P ≤ 0.001) and from 28.6 to 31.7% (^***^P ≤ 0.001), respectively. Whereas the use of laser significantly increased 4T1 cells death and induced apoptosis from 29.2 to 42.8% (Q2) and from 31.7 to 33.4% (Q3). In this regard, our results in Fig. 3E confirmed the toxicity of FeS-Dox@bLf NZs compared to the free Dox by increasing the intracellular ROS. It can also be seen that the ROS level in the use of laser on the platform of FeS-Dox@bLf NZs is almost 3.03 folds than that of the control group, 1.84 times that of free Dox and 1.35 folds than that of FeS-Dox@bLf NZs.

### In vivo assays

**Fig. 4:**
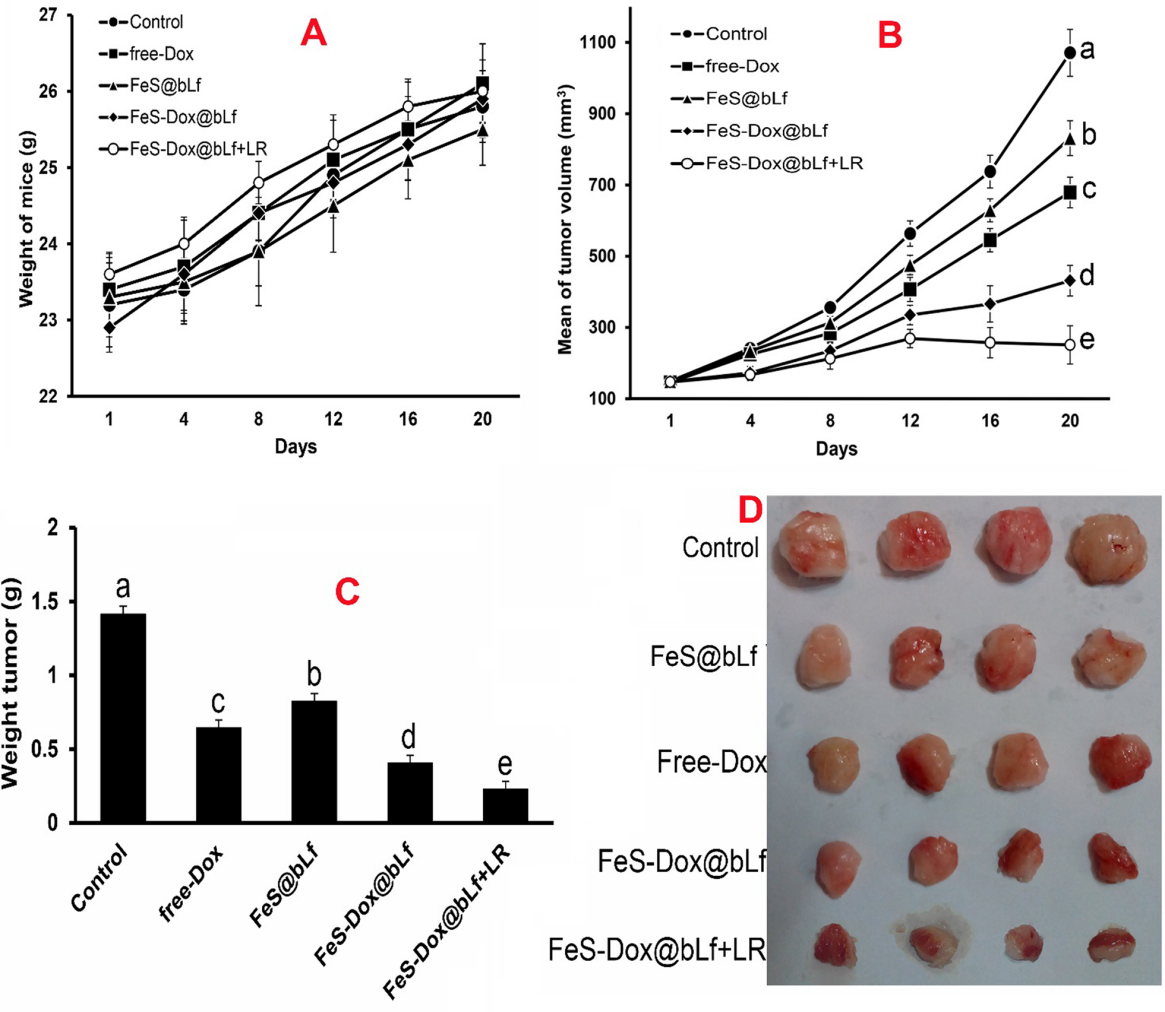
**A** Body weight change of mice. **B** Tumor volume curves of different groups after treatment. **C** Mean weight, and (**D**) digital photographs of tumor on day 20. ^a,b,c,d,e^LS means with different letters in superscripts are different at ^*^P < *0.05*

Because the results of in vitro method were considered favorable, cancer mice were used to evaluate the therapeutic effects of FeS-Dox@bLf NZs with or without laser irradiation in vivo condition. There was no significant difference in the weight of mice during the experimental period (Fig. 4A). However, compared to other groups, control mice had a higher rate of tumor growth. The results of Fig. 4B revealed that the integration of chemotherapy with gas therapy by FeS-Dox@bLf NZs causes a significant difference compared to the control and free Dox groups. In this regard, the use of laser irradiation on the platform of FeS-Dox@bLf NZs according to raising ROS increased the efficiency of treatment and significantly reduced the volume of breast tumors compared to free Dox and even FeS@bLf NZs. Furthermore, the results of Fig. 4C, D confirmed that the combination of chemotherapy and PDT reduces tumor volume by up to ~ 6.2 fold than the control group, ~ 2.84 fold than the free Dox and ~ 1.77 times the FeS-Dox@bLf NZs at the end of the experimental period.

**Fig. 5:**
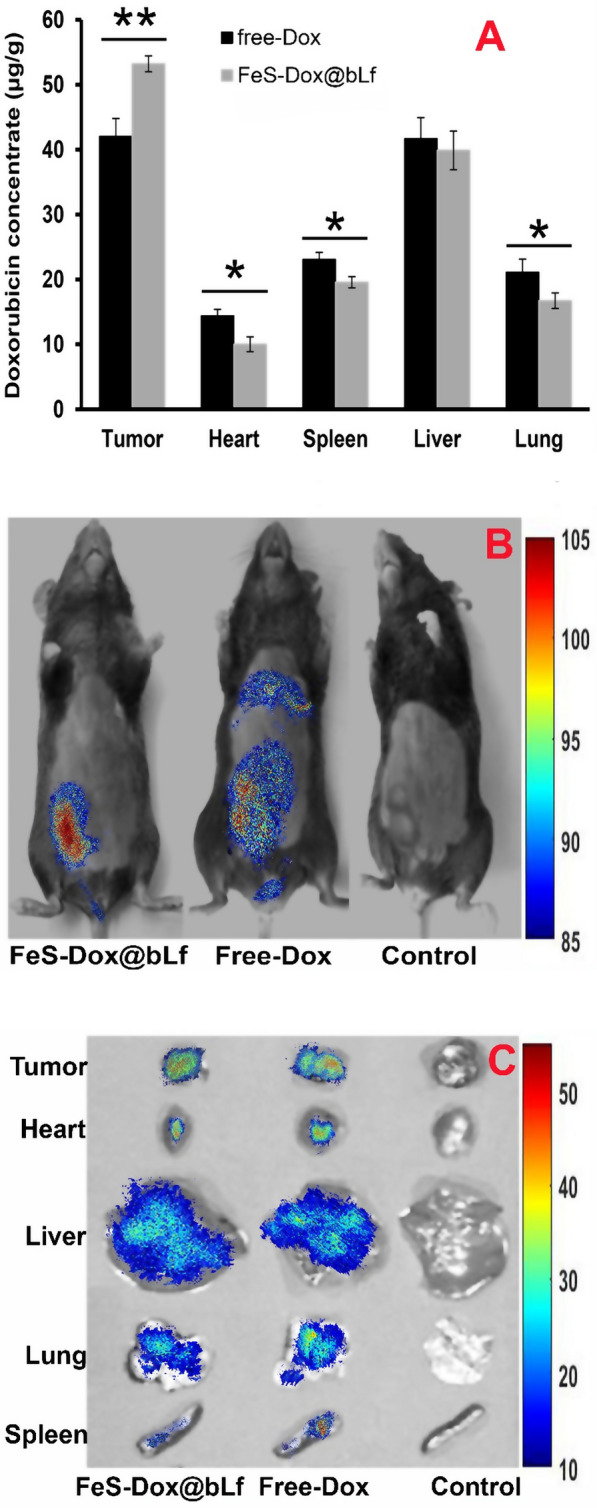
**A** Concentration of Dox in tumor and major organs. **B** Biological distribution of Dox in whole body, and (**C**) Major organs in mice by fluorescence imaging, 8 h after they were i.v. injected with free Dox, and FeS-Dox@bLf NZs. Statistical differences were measured at level of ^*^P < *0.05*, and ^**^P < *0.01*

As shown in Fig. 5A, Dox accumulation by FeS-Dox@bLf NZs was significantly increased in tumor tissue compared to free Dox. The use of nanocarriers increased drug delivery from non-target tissues to the tumor, and it significantly reduced drug levels in heart, spleen and lung tissues. Likewise, the fluorescence intensity results in Fig. 5B revealed that Dox-wide distribution throughout the whole body using the FeS-Dox@bLf NZs focuses on tumor tissue. While the distribution of free Dox throughout the body is more widespread. On the other hand, the intensity of fluorescent in vital organs shows that the use of FeS-Dox@bLf NZs increases the intensity of fluorescent in tumor tissue and decreases the intensity of fluorescent in the organs of the heart and spleen (Fig. 5C). However, no significant difference in fluorescent intensity was observed in liver and lung tissue. Therefore, the use of FeS-Dox@bLf NZ can reduce the toxicity of Dox in heart tissue, which is very undesirable for the heart in chemotherapy. Overall, the results of Fig. 5 show the successful targeting of nanoplatform-based drugs.

**Fig. 6:**
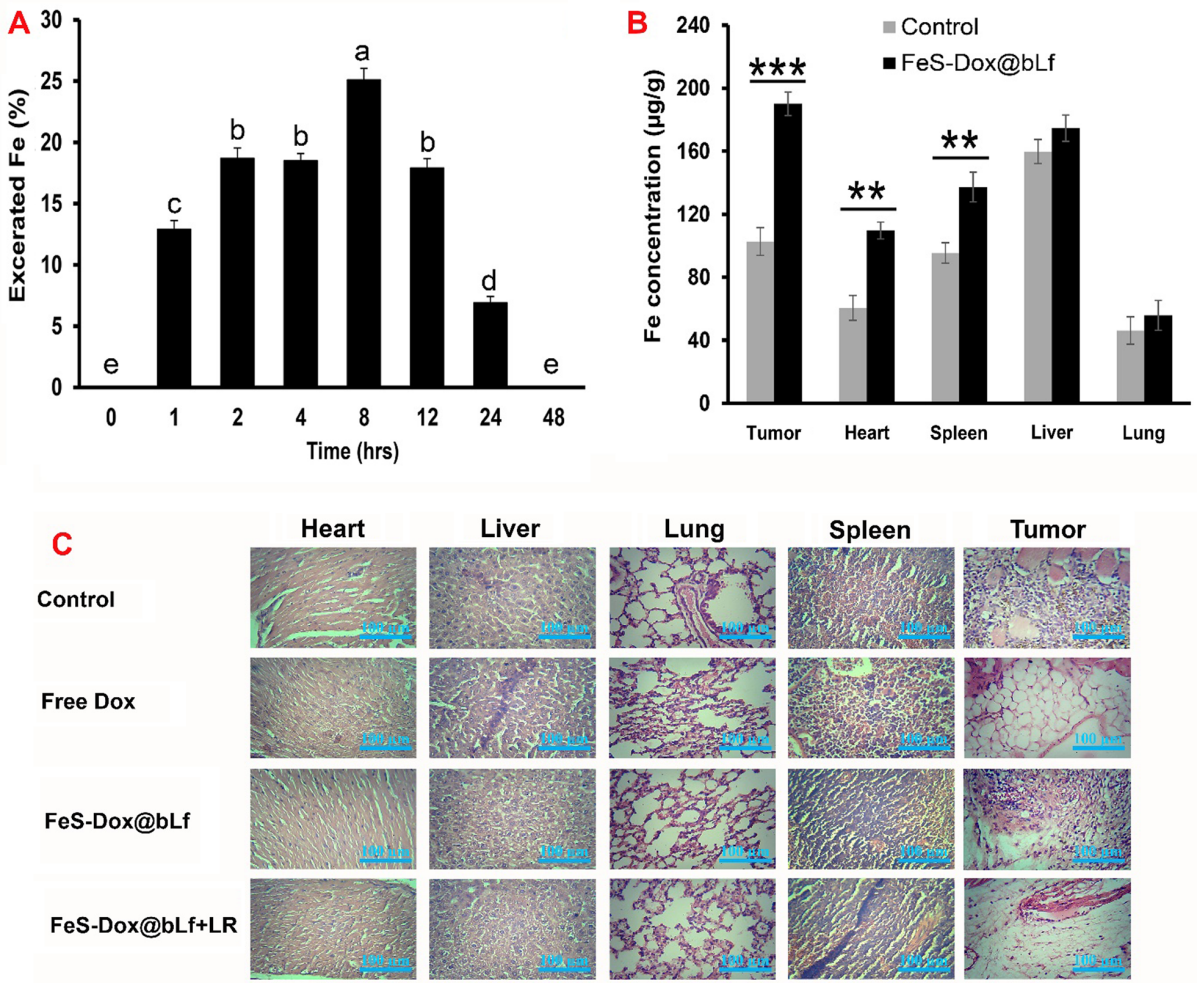
**A** The content of Fe element in mice urine and faces after injection of FeS-Dox@bLf. **B** Biological distribution of Fe in major organs, and (**C**) Histological observation of treated breast tumor tissues and major organs include of heart, lung, liver and spleen visualized using H&E staining. ^a,b,c,d,e^LS means with different letters in superscripts are different at ^*^P < *0.05*. Statistical differences were measured at level of ^**^P < *0.01*, and ^***^P < *0.001*

In addition, to evaluate Fe of urine plus faces, the results of Fig. 6A show that the injected NZs can be excreted from the body within 24 h, considering the amount retained in the tissues of the spleen, lungs and heart. This result therefore explains that the amount of Fe received from the body is excreted and the level of toxicity is greatly reduced. In the following, the results of Fig. 6B showed an increase in Fe accumulation in tumor tissue relative to the control sample.

Finally, a histological method was used to evaluate cytotoxicity in the liver, heart, lung and spleen organs. As shown in Fig. 6C, the use of free Dox causes adverse changes in heart tissue. While the use of FeS-Dox@bLf NZs with and without irradiation laser shows, the adverse effects of the drug on heart tissue are greatly reduced. It was also found that the use of different treatments did not cause significant damage to liver, spleen and lung tissue. However, analysis of tumor tissues reveals that the most important morphological changes in 4T1 tumor cells are caused by FeS-Dox@bLf NZs + Laser. However, changes in tumor tissue in the FeS-Dox@bLf NZs group versus free Dox are significant and remarkable. Staining results showed that the tumor tissue structure had disappeared in free Dox, FeS-Dox@bLf NZs with and without laser irradiation, and that some of the remaining breast cancer cells had shrunk (Fig. 6C). Typically, cancer cells appear brown with a state of necrosis or apoptosis, and live cells appear pink.

## Discussion

Nowadays, the use of magnetic NPs with protein coating to target in medical activities due to the ability to synchronize an auxiliary’s therapies such as PTT, PDT, with chemotherapy are highly regarded [37–39]. Therefore, to control and treat breast cancer, we designed relatively uniform FeS NZs based on the results of the SEM, TEM and DLS (Fig. 1), which was confirmed by the FT-IR results. The physicochemical properties of FeS NZs were consistent with the findings of Sathiyaraj and Thirumaran [40] and Yang et al. [41]. On the other hand, the results of SEM, TEM and FTIR similar to the outcomes of Shankaranarayanan et al. [42] and Sharifi et al. [8] showed that bLf and Dox were loaded on FeS NZs.

Metallic NPs such as Fe, manganese (Mn), copper (Cu), etc. as NZs are able to perform chemical reactions in biological solutions [9, 43, 44]. On the other hand, it has been confirmed that amorphous metallic NZs especially Fe NZs perform better Fenton reactions than bare NZs due to higher ionization and release of metal ions [45]. Therefore, parallel to the activities of He et al. [46] who showed that NH2-MIL-88B(Fe) NZs change the color of MB by producing ^•^OH caused by Fenton reactions, in this study it was found that FeS@bLf NZs over time cause the digestion of MB in the presence of H_2_O_2_ and discoloration (Fig. 2A). Although the study of the MB degradation by FeS-Dox@bLF NZs in light without dark findings is a limitation in this paper, according to the findings of Molla et al. [47] who revealed that using visible light against darkness can increase the rate of MB degradation, it can be confirmed that the generated FeS-Dox@bLF NZs had a favorable catalytic effect in the decomposition of MB. Therefore, this procedure can provide a faster response for the researcher. But, in future research, the effect of FeS-Dox@bLF NZs on the decomposition of MB in light and dark can be performed to provide greater assurance of the activity of NZs to produce radicals in vivo. However, this experiment shows that the produced FeS-Dox@bLF NZs will have good catalytic activity, especially during laser irradiation, which is relatively similar to the state of light irradiation. Digestion of MB in the presence of FeS-Dox@bLf indicates the peroxidase-like activity of NZs with the release of Fe^2+^, which was in agreement with the finding of Xie et al. [48]. Furthermore, the outputs of this report (Fig. 2A) similar to the results of Maji et al. [49] exhibit that the enzyme-like activities of FeS@bLf NZs are highly dependent on the duration of the presence of NZs, such as concentration, crystalline nature and particle size of NZs [50, 51].

Since it has been proven that the release of metal ions increases their enzyme-like activity [50], inducing the release of Fe^2+^ in cancerous cells increases the possibility of therapeutic activities. In this regard, in accordance with the results of Xie et al. [48], this study revealed that FeS-Dox@bLf NZs release Fe^2+^ in acidic conditions in a favorable manner, which has been significantly effective in therapeutic activity (Fig. 2B). Likewise, He et al. [26] explained that MnS@BSA NZs increased enzyme-like activity by releasing Mn^2+^ under acidic conditions. In this regard, the H_2_S release at pH 6.5 indicated an increase in the release of Fe^2+^ along with S^2−^ from FeS-Dox@bLf NZs (Fig. 2C). Similar to our findings, Ma et al. [52] and Kou et al. [53] explained that release of Cu^2+^ and Fe^2+^ from the metal–organic framework and Fe@carboxymethyl cellulose NPs, respectively, increase gas release in acidic condition compared to neutral condition. Moreover, comparable to the results of other findings [26, 48], this report suggests that the use of protein coatings such as bLf delays the burst release of H_2_S to further load NZs into cancer cells. Along with Fe^2+^ release, releasing H_2_S from FeS-Dox@bLf NZs in acidic condition is more than neutral, which can be very important in cancer treatment activity due to the tumor microenvironmental acidic conditions. In addition to the release of Fe^2+^ and H_2_S, Dox loaded on FeS-Dox@bLf NZs will be very effective on therapeutic activity. In agreement with the results of Singh et al. [54], Sharifi et al. [6], and Sharifi et al. [8] Table 1 shows that the release of Dox from FeS-Dox@bLf NZs increases with decreasing environmental acidity. Analogous to the release of Fe^2+^ and H_2_S, the mechanism of release of Dox from FeS-Dox@bLf NZs into cells can be related to the effect of lysosome activity, the acidic condition of the tumor microenvironment, and endosome activity.

The toxic effects of FeS-Dox@bLf NZs with and without laser irradiation in Fig. 3A indicated that their negative effects on 4T1 cancer cells are dose-dependent. This finding, together with the results of the effect of NZs on the increase of ^•^OH and H_2_S through Fe^2+^ catalytic activity (Fig. 3B), are in agreement with the results of Xie et al. [48] and Xiao et al. [55], which generally all point to the induction of apoptosis via ROS with intracellular biological changes such as DNA damage and mitochondrial dysfunction [56]. Although the lethal level of H_2_S on 4T1 cancerous cells is less important than ^•^OH, H_2_S induces apoptosis by increasing the intracellular ROS through the Fenton reaction of Fe^2+^ due to the cessation of catalase activity [57] and excessive accumulation of H_2_O_2_ [58]. Moreover, in line with the result of Chang et al. [59], this study revealed that the synergy of chemotherapy with PDT increases intracellular ROS and apoptotic cellular death.

In vivo evaluations in this study (Fig. 4) are similar to the studies of She et al. [60] and Xie et al. [48] confirming the significant effects of FeS-Dox@bLf NZs on the size and volume of breast tumors. Numerous anti-tumor activities of drug-containing Fe NPs in breast cancer have been published parallel to this report [6, 8, 61–63], which in addition to reducing drug resistance through intelligent drug delivery increased the performance of anti-cancer activity by synchronizing PTT or PDT. Increased performance of FeS-Dox@bLf NZs is not only related to PDT to induce apoptosis through H_2_S, but also the results of Fig. 5 show that increasing the load of Dox via FeS-Dox@bLf NZs on breast tumors indicates that they are more effective in treating breast cancer. Consistent with this finding, Sharifi et al. [6] and Sharifi et al. [8] showed that Dox focused more on breast cancer tissue using Fe NPs compared to the control group. However, the positive effect of using PDT on metallic NZs containing drugs in tumor therapy is similar to the finding of Fang et al. [64] who showed that the effect of gas therapy to reduce drug resistance is very significant.

Subsequently, in vivo evaluation based on the results of Fig. 5A demonstrated that Dox accumulation was significantly reduced in non-target tissues, especially in the heart, by targeting the drug with FeS-Dox@bLf NZs, which was in line with the outcomes of Tang et al. [65] and Wang et al. [66]. This reduction significantly eliminates the side effects of chemotherapy. Meanwhile, Sharifi et al. [8] explained that the use of bLf coating increases the drug loading level in breast cancer tissue by twice to three times. Lack of accumulation of Fe NPs in lung and liver tissues, and excessive increase in Fe accumulation in tumor tissue can confirm the accumulation of Dox in tumor tissue. As well, the removal of Fe NPs from the body within 48 h (Fig. 6A) analogous to the result of Mn rapid clearance from the body of mice described by He et al. [26] indicates a reduction in the severity of toxicity of the use of FeS in long-term activity. In another part of the study, comparable to the reports of Sharifi et al. [6] and Luo et al. [67], it was shown (Fig. 6C) that the synchronization of PDT with chemotherapy significantly destroys the cellular structure of breast tumors and enhances therapeutic activity.

## Conclusions

In general, a new type of amorphous FeS-Dox@bLf NZs sensitive to environmental acidity was designed by a wet-chemical method that has the ability to synchronize chemotherapy and PDT to enhance H_2_S production for the treatment of breast cancer. During synthesis, it was determined that FeS-Dox@bLf NZs show peroxidase-like activity in acidic condition by releasing Fe^2+^ and S^2−^ to produce ^•^OH and H_2_S, respectively. Therefore, the use of FeS-Dox@bLf NZs in the treatment of breast cancer with relatively acidic microenvironments can be beneficial by increasing free radicals and gas. For this purpose, evaluations of intracellular ROS and their effects on apoptotic cellular death confirmed the effects of FeS-Dox@bLf NZs for the treatment of 4T1 cancerous cells, which was enhanced by the synchronization of chemotherapy and PDT. Furthermore, FeS-Dox@bLf NZs with targeted transfer and further release of Dox on breast tumors based on the tumor microenvironment acidic condition have allowed the drug to be loaded, which causes less toxicity in other tissues, especially in the heart. In vivo evaluations in this study also revealed that FeS-Dox@bLf NZs are highly biocompatible for long-term treatments due to the removal of Fe from the body within 48 h and the concentration of FeS-Dox@bLf NZs on the target tissue. Overall, all the in vitro and in vivo observations determined that FeS-Dox@bLf NZs are not only successful in treating and controlling breast cancer with minimal side effects, but also it provided a clear way to synchronize different therapies such as chemotherapy, PDT, and so on.

## Declarations

### Availability of data and materials

The datasets used and analysed during the current study are available from the corresponding author on reasonable request.
